# Relationship between unmet needs for assistance and healthy aging among disabled older adults in China

**DOI:** 10.3389/fpubh.2022.914313

**Published:** 2022-07-20

**Authors:** Yang Cao, Yuxin Feng, Yaling Luo

**Affiliations:** Department of Labor and Social Security, School of Public Administration, Sichuan University, Chengdu, China

**Keywords:** unmet needs, health, activities of daily living, cognition, participation

## Abstract

**Background:**

Although there is a growing consensus around the world that long-term care services and supports are important to help the aged population with disabilities achieve healthy aging, a misallocation of care resources and inefficiency in care delivery still exist in China. The absence or inadequate provision of long-term care services and supports among older adults with disabilities results in a range of adverse health consequences. However, the negative influence of unmet needs for assistance on healthy aging, based on functional perspectives including physiological, psychological, and societal domains, has been underestimated. This study aimed to measure healthy aging based on a person-centered approach and examine the relationship between unmet needs for assistance and healthy aging among older adults with disabilities in China.

**Methods:**

Based on the data from the Chinese Longitudinal Healthy Longevity Survey 2018, we used the latent profile analysis with three indicators to uncover distinctive types of older adults experiencing distinct levels of healthy aging, and applied the ordered logit regression to analyze the correlation between unmet needs for assistance and different levels of healthy aging. To further address the endogeneity bias, the robust test was conducted by the two-stage least-squares instrumental variable estimation and the conditional mixed process instrumental variable estimation.

**Results:**

Three ordered latent classes were identified: a low level of healthy aging (42.83%), a middle level of healthy aging (47.27%), and a high level of healthy aging (9.90%). Disabled older adults with unmet needs had a lower probability of achieving the higher level of healthy aging (*OR* = 0.57, *SE* = 0.04, *CI* = 0.48–0.66, *p* < 0.001).

**Conclusions:**

This study highlights the need to increase awareness among gerontological practitioners with respect to long-term care services and supports for disabled older adults as a potential for enhancing their healthy aging, and that unmet needs could be a basis for risk assessment and a means for determining the efficacy of long-term care interventions on maintaining health.

## Introduction

As a response to the expansion of life expectancy but the increase of years lived in poor health over the past three decades (1), policy decisions concerning ways to advance healthy aging have become part of health and social agendas for many countries. However, there is some difficulty in developing policy aimed at advancing healthy aging while there remains confusion as to the definition and measurement of the concept itself. Existing studies created different operational definitions of healthy aging from more to less rigid and identified the respondents experiencing healthy aging by using different definitions. They found that the use of definitions that emphasize functional limitations is more useful in distinguishing the truly healthy from the truly unhealthy than the use of ones that are based on rigid disease criteria (2). Therefore, although there appeared to be no agreed standard by which aspect of healthy aging could be measured, there was consensus in the studies that the multidimensional and positive health outcome should be used to capture the capacity to function well and adapt to environmental challenges, including assessing physical functioning, most frequently a measure of activities of daily living (ADLs), mental functioning, most frequently a measure of cognition, and social functioning, most frequently a measure of social participation (3, 4).

There is a growing consensus around the world that long-term care (LTC) services and supports are important to help the aged population with disabilities achieve healthy aging under the new concept of healthy aging proposed by the World Health Organization (WHO) (5). The provision of continuous and adequate LTC services and supports for older adults in need can help them maintain the highest possible quality of life with the greatest possible degree of independence, autonomy, participation, personal fulfillment, and human dignity (6). In China, 35 cities in 14 provinces have begun to launch LTC insurance programs as pilots toward a nationwide LTC insurance program since 2016, and the number of pilot cities has been expanded to 49 since 2020. Although LTC services and supports have developed since the 2010s in China, there is still a misallocation of care resources and inefficiency in care delivery. As of 2018, nearly 3% of community-living Chinese older adults with disabilities reported that they did not receive any help with ADLs, defined as completely unmet needs, and over 50% of them reported that the assistance they received did not fully meet their needs, defined as under-met needs (7). More rural older residents experienced completely unmet needs than urban residents (8). From 2005 to 2014, the proportion with completely unmet needs almost doubled for all disabled older adults (7).

The absence or inadequate provision of assistance with daily living tasks among older adults with disabilities can result in a range of adverse consequences. Prior research used descriptive statistics to report that the prevalence of people experiencing adverse consequences, such as not being able to bathe or shower, not being able to put on clean clothes, falling out of bed or a chair, wetting or soiling oneself, or going hungry, was greater for those with unmet vs. met needs (9, 10). Other longitudinal studies focusing on the adverse health-related consequences reported that unmet needs for assistance might increase the risk of death (11, 12), hospitalizations (13, 14), and rehospitalizations (15, 16), and also exacerbate depressive symptoms (17). Deterioration in health in turn will increase service uses and the risk of experiencing unmet needs (18, 19). As thus, the provision of sufficient LTC services and supports is a fundamental social intervention to achieve healthy aging.

Existing research focused on the impact of unmet needs for assistance on the biomedical level of health while overlooked its influence on the psychosocial perspective, which is a crucial component of healthy aging. And thus, the adverse health-related consequences of unmet needs might be underestimated. Contrary to the traditional biomedical model, the biopsychosocial model and the new concept of healthy aging emphasize the maintenance of functioning, which is determined by assessing physical and mental capacities and the interactions with the environment (5, 20). It is necessary to consider multiple indicators of healthy aging at the same time because there are often reciprocal relationships between functional limitations, cognitive impairment, and social participation, which result in clustering of personal features. However, ADLs and cognitive function, the main indicators of physical and mental capacities (5), have been overlooked in the studies of adverse consequences of unmet needs. In addition, the relationship between unmet needs and participation, an indicator which has been defined as the observable performance within a life situation and viewed as a societal perspective of functioning (21), has not been examined either.

The disablement process model proposed by Verbrugge and Jette elucidated the causal process from disease to disability, which indicated that adequate personal assistance provided for people during the disablement process could reduce their difficulties in performing expected or specified social role activities (22). Grossman's framework of a health production function also pointed out that individuals' initial stock of health tended to depreciate with aging, but could be enhanced by investments in oneself over time (23). LTC services and supports are important investments in maintaining and improving the performances of ADLs among older adults with disabilities. And thus, disabled older adults with unmet needs for assistance would experience more severe impairments in ADLs than those with met needs.

Basic mental actions include central cognitive and emotional functions (22). Prior research showed an adverse consequence of unmet needs for emotional functions (17). Although there is no direct evidence for the correlation between unmet needs for assistance and cognitive function, a few studies reported that larger social networks or more social supports were associated with better cognitive function among older adults (24–28). Considering that unmet needs for assistance indicate the absence or inadequate provision of LTC services and supports provided by formal or informal caregivers, disabled older adults with unmet needs for assistance may experience more severe functional limitations in cognition than those with met needs.

A few studies have shown that adequate social supports have a positive influence on participation in various activities among older adults and patients with chronic diseases (29–32), but very few directly examined the impacts of unmet needs for assistance on participation among disabled older adults. Chong et al. (33) indicated that unmet needs for home and community-based services were associated with a lower likelihood of being active in the community and interacting with families and friends among Medicaid users (33). Since older adults with disabilities have reduced levels of social participation, adequate assistance and supports are prerequisites for them to participate in community and social activities (34), which are further associated with better health and social well-being (35–38).

As the definition of healthy aging ranged from a primarily biological model to a comprehensive biopsychosocial model, most recent studies emphasized the maintenance of functional independence in their definition and measured healthy aging based on physiological, psychological, and societal domains. Although existing studies used national survey data to estimate the prevalence of healthy aging, cut-off scores on the measures selected were based on the distribution in the study population and to capture those functioning above the median (39), in the top tertile (40), quartile (41), or quintile (42). WHO theoretically divided the trajectory of functional ability into three periods, including a period of relatively high and stable capacity, a period of declining capacity, and a period of significant loss of capacity (5), but little empirical research has been conducted to distinguish healthy aging into such different levels. In order to fill the above research gaps and inform policy development, this study used the national data to generate the typology of older persons experiencing different levels of healthy aging based on a person-centered approach, and thus proposed the first hypothesis: Older adults would be classified as experiencing a low level, a middle level, and a high level of healthy aging, respectively.

Previous studies have established the relationship between unmet needs and a set of single health indicators, mainly representing the biomedical level of health (11–17), but little is known about whether the absence or inadequate provision of LTC services and supports will prevent the aged population with disabilities from achieving a higher level of healthy aging. Understanding the relationship between unmet needs for assistance and different levels of healthy aging can provide evidences for incorporating unmet needs into the need assessment and establishing a precise LTC service supply and subsidy mechanism based on unmet needs, and also for developing an alternative health intervention that regards unmet needs as a risk factor. To fill the research gaps, we further examined the correlation between unmet needs for assistance and different levels of healthy aging among Chinese older persons with disabilities, and thus propose the second hypothesis: Disabled older adults with unmet needs for assistance would have a lower probability of achieving a higher level of healthy aging than their counterparts with met needs.

## Methods

### Data source and samples

In this study, we employed data from the Chinese Longitudinal Healthy Longevity Survey (CLHLS), a nationwide survey that collected data on both the extent to which disability-related needs were met and healthy aging among the Chinese aged population. CLHLS is a nationally representative survey of the older population aged 65 or older from 23 (out of 31) provinces in China, with an oversampling of the oldest-old aged 80 and older (43). CLHLS has 8 waves (1998, 2000, 2002, 2005, 2008, 2011, 2014, and 2018), and survey respondents were randomly chosen from half of the counties and cities in the 23 selected provinces, where the population constituted 85% of the total population in China (44). Zeng described in detail the survey design, sample distribution, and data quality of the CLHLS (44). This study employed the Wave 8 survey data because it is the latest wave.

The 2018 follow-up survey contains 15,779 respondents aged 65 years and above. Considering that this study focused on ADL care needs and the analysis of unmet needs should target the population with care needs, the study sample excluded 11,030 (69.90%) respondents who reported having no disabilities, defined as having no limitations in six ADLs. Among the 4,749 respondents with disabilities who had at least one ADL limitation, 1,870 (39.38%) of them had missing data on dependent variables, independent variables, or control variables. After excluding respondents with missing data, the final analytic sample included 2,879 older adults with disabilities.

### Measures

#### Dependent variables

The healthy aging level was the dependent variable in this study. According to the definition of healthy aging, we selected three indicators to generate the typology of older persons experiencing different levels of healthy aging, including ADLs, cognition, and participation.

ADLs were evaluated by Katz's index of ADLs, which has been demonstrated as a reliable scale to assess ADLs (Cronbach's a = 0.75) (45). The index consisted of six items including bathing, dressing, toileting, eating, indoor transfer and continence. Regarding each activity, respondents were asked “do you require assistance when performing each activity (e.g., eating).” The answer option that they performed the activity by themselves was coded two. The answer options that they required some or complete help with that ADL were coded one or zero, respectively. As such, the total score ranged from 0 to 12, with an equally weighted sum of the six items. Since respondents without ADL limitations were excluded from the final analytic sample, the total score of ADLs ranged from 0 to 11, with a higher score indicating fewer ADL limitations. ADLs, cognition, and participation were measured on different scales, so z-scores were calculated by subtracting the mean score of study sample from the original score and then dividing the standard deviation, so as to make them more comparable when generating the typology of older persons experiencing different levels of healthy aging.

Cognition was measured by the Mini-Mental State Examination scale (MMSE) (46) with a Cronbach's alpha value of 0.97. The scale consisted of twenty-four items to test five domains of cognitive function, which included abilities of orientation, registration, attention and calculation, recall, and language. The total score ranged from 0 to 30, with a higher score indicating better cognitive function. We used the z-score of cognition to generate the typology of older persons experiencing different levels of healthy aging.

Participation has been usually measured by the performance in a standard set of roles and activities. For people with disabilities, domestic life (including household tasks, caring for household objects, etc.) and community and social life (including recreation and leisure, community life, etc.) are important participation domains, which can help slow functional decline (21, 35). Thus, participation in this study was measured by the frequency of involvement in domestic activities (including housework, garden work, and raising domestic animals) and community and social activities (including personal outdoor activities, reading newspapers/books, playing cards/mah-jong, watching TV/listening to the radio, organized social activities). For each of the eight activities, respondents were asked “do you perform each activity regularly (e.g., housework).” The response options, “almost every day,” “at least once a week,” “at least once a month,” “not every month,” or “never,” were coded 4, 3, 2, 1, and 0, respectively. As such, the total score of all the activities ranged from 0 to 32, with a higher score indicating higher participation. Additionally, we used the z-score of participation to generate the typology of older persons experiencing different levels of healthy aging.

#### Independent variable

Informed by previous studies (7, 19), unmet needs for assistance with ADLs, a dummy variable, was selected as the independent variable in this study. Respondents were asked “who is the primary caregiver when you require assistance with the ADLs?” Respondents choosing “nobody” were considered to have a completely unmet need for assistance, while others choosing any caregiver (including spouse, son, daughter-in-law, daughter, son-in-law, unmarried son and daughter, grandchildren, other relatives, neighbors, social service, and housemaid) were further asked “did the help that you received with the ADLs meet your needs?” Respondents who reported that the assistance they received only met part of their needs or did not meet them at all were considered to have an under-met need for assistance, while those who reported having their needs fully met were considered to have a met need for assistance. Respondents who experienced an under-met need or completely unmet need were considered to have an unmet need.

#### Control variables

Demographic characteristics, socioeconomic status, health status and behaviors, and availability of medical insurance and services might confound the relationship between unmet needs and different levels of healthy aging (23, 47), and have been controlled in the analyses. Demographic characteristics included age (in years), gender (female = 1), marital status (currently married = 1), rural-urban residence (urban residence = 1), and geographic area (east =1, central =2, west =3). Socioeconomic status was measured with education level (educated = 1) and self-reported sufficiency of income (had sufficient income = 1). Health status and behaviors were measured by chronic illnesses, smoking, drinking, and doing exercises, all of which were dummy variables. Availability of medical insurance was coded one when respondents reported that they had medical insurance, otherwise, coded zero. Availability of healthcare services was coded one when respondents reported that they received adequate healthcare services when they were sick, otherwise, coded zero.

### Data analysis

First, we used descriptive statistics to report the characteristics of the study sample. To explore ordered latent classes of older adults experiencing different levels of healthy aging, we conducted latent profile analysis (LPA) with three observed standardized indicators, including ADLs, cognition, and participation. LPA is a statistical method that identifies unobserved classes within a population based on responses to a set of observed continuous variables (48). The number of latent classes is determined by a variety of statistical indices, including Akaike's Information Criterion (AIC) (49), Bayesian Informal Criterion (BIC) (50), adjusted BIC (aBIC) (51), Entropy, and the *p*-value of the Lo-Mendell–Rubin Likelihood Ratio Test (LMR-LRT) (52). Better model fit is determined by lower values of AIC, BIC, and aBIC, and better separation of latent classes is indicated by higher Entropy values (53). Besides, the significant *p*-value of the LMR-LRT indicates that the model with k classes fits the data better than the more parsimonious model with k-1 classes (53). There are two parameters estimated with LPA that are important for this study. The first is the mean of each indicator, which endorse each observed indicator within a given latent class and are used to interpret and label each identified class. The second parameter is the probability of class membership, which describes the probability distribution of the latent classes, which sum to one. Overall, model fit, parsimony, interpretability of latent classes, and probabilities of class membership determines the optimal model (54).

Second, the ordered logit regression model, appropriate for ordinal-category outcome variables, was estimated to test the association between unmet needs for assistance and different levels of healthy aging, while controlling for confounding variables. However, the relationship between unmet needs and healthy aging might be spurious for two reasons. First, reverse causality might exist due to the simultaneous determination of these variables. Second, although we have controlled for many potential confounders, there might be unobserved heterogeneity due to omitted variables. In order to address these endogeneity problems, this paper further used the two-stage least squares (2SLS) model, appropriate for continuous outcome variables, and the conditional mixed process (CMP) model, appropriate for both continuous and categorical outcome variables, to conduct the instrumental variable estimation. The provincial old-age dependency ratio, calculated by the ratio of the number of people aged 65 or above to that aged 15–64 in each province, was utilized to construct the instrumental variable, because it was not directly correlated with individuals' health conditions but showed a strong correlation with unmet needs with the *F* value of 15.33 in the weak instrumental variable test. The LPA models were estimated with Mplus, version 8.3, and other analyses were performed using Stata, version 15.1.

## Results

### Sample characteristics

Table 1 shows the characteristics of the study sample. Around half of the respondents had unmet needs for assistance. The average age of the respondents was 95 years old. About 70% of the respondents were female. Less than 20% of the respondents were currently married. Sixty perecent of the sample individuals resided in urban areas. More than half of the respondents lived in eastern areas. More than 30% of the respondents ever received education. More than 80% of them reported sufficient income. 74% of the respondents suffered from at least one chronic disease. Less than 10% of the respondents smoked or drank wine. The number of the respondents taking exercises accounted for no more than 15%. More than 80% of the respondents had medical insurance, and around 95% of the sample individuals received adequate healthcare services when they were sick.

**Table 1:** Sample characteristics (Means/Proportions) (*N* = 2879).

**Variables**	**Mean (SD)/N (%)**	**Variables**	**Mean (SD)/*****N*** **(%)**
Unmet needs for assistance	1452 (50.43%)	**Health status and behaviors**	
**Demographic characteristics**		Had chronic illness	2132 (74.05%)
Age	94.95 (8.44)	Smoked	217 (7.54%)
Female	1929 (67.00%)	Drank	200 (6.95%)
Currently married	462 (16.05%)	Took exercises	364 (12.64%)
Urban residence	1747 (60.68%)	**Availability of medical insurance and services**	
**Geographic area**		Had medical insurance	2367 (82.22%)
East	1605 (55.75%)	Received adequate healthcare services	2751 (95.55%)
Central	746 (25.91%)	**Indicators of healthy aging**	
West	528 (18.34%)	ADLs	7.05 (3.36)
**Socioeconomic status**		Cognition	14.30 (10.53)
Educated	935 (32.48%)	Participation	3.00 (3.98)
Had sufficient income	2423 (84.16%)		

### Indicators and levels of healthy aging

Table 1 also presents the distribution of three healthy aging indicators. The average score of ADLs was 7.05 among respondents. Respondents' mean value of cognition score was 14.30. The average score of participation was 3.0.

The model-fit statistics for the LPA models with one to four classes are presented in Table 2. The LMR-LRT showed that the four-class model was not significant, suggesting that the three-class model fit was better than the four-class model. In addition, the three-class model yielded a better model fit than the two-class model, because the values of AIC, BIC, and aBIC of the three-class model were lower than those of the two-class model. Given these model parameters, we selected the model containing three latent classes.

**Table 2:** Comparison of fit statistics for LPA models with one to four classes (*N* = 2879).

**Number of Classes**	**AIC**	**BIC**	**aBIC**	**Entropy**	**LMR test (p)**
1	24519.744	24555.535	24536.471	/	/
2	22457.536	22517.188	22485.415	0.861	0.0000
3	21569.226	21652.739	21608.256	0.862	0.0113
4	21177.401	21284.775	21227.582	0.883	0.5147

The parameters for the three-class model are presented in Table 3. The prevalence of the three latent classes and the average z-scores of each indicator were reported under each class. The most common class of healthy aging was a middle level of healthy aging (47.27%), characterized by moderate mean values on z-scores of ADLs, cognition, and participation. The second most common class was a low level of healthy aging (42.83%), characterized by the lowest average z-scores on all the indicators. The least common class was a high level of healthy aging (9.90%), characterized by the highest average z-scores on all the indicators. Overall, the above results supported the first hypothesis.

**Table 3:** Three-class model of healthy aging levels among Chinese older adults with disabilities (*N* = 2879).

	**Low level of healthy aging**	**Middle level of healthy aging**	**High level of healthy aging**
*Latent class prevalence*	42.83%	47.27%	9.90%
*Item average z–scores (SE)*			
ADL	−0.53^***^(0.03)	0.33^***^ (0.03)	0.77^***^ (0.03)
Cognition	−1.00^***^(0.02)	0.70^***^ (0.03)	1.06^***^ (0.04)
Participation	−0.50^***^(0.02)	0.002^***^ (0.05)	2.20^***^ (0.21)
*Defining characteristics*	lowest average z–scores on all the indicators.	moderate average z–scores on all the indicators.	highest average z–scores on all the indicators.

*** *p < 0.001*.

### Unmet needs and healthy aging levels

#### Ordered logit regression analysis results

The regression model findings for unmet needs and healthy aging levels were summarized in Table 4. We used the “met needs” as the reference group in the regression analysis for healthy aging levels. To ease interpretation, we reported odds ratios for the independent and control variables. Compared to those with met needs for assistance, disabled older adults with unmet needs had a lower probability of achieving the higher level of healthy aging (*OR* = 0.57, *SE* = 0.04, *CI* = 0.48–0.66, *p* < 0.001), which supported the second hypothesis. Regarding control variables, disabled older adults, who were younger, married, better educated, lived in urban or eastern areas, received sufficient income, suffered from chronic illness, smoked, drank, took exercises, or received adequate healthcare services, had a higher probability of achieving a higher level of healthy aging.

**Table 4:** Summary of results from regression analysis of unmet needs and healthy aging levels among Chinese older adults with disabilities (*N* = 2879).

	**Odds ratio (SE)**	**95% Confidence interval**
Unmet needs	0.57^***^ (0.04)	0.48–0.66
Age	0.93^***^ (0.01)	0.92–0.94
Female	1.08 (0.10)	0.90–1.30
Currently married	1.35^*^ (0.16)	1.06–1.71
Urban residence	1.26^**^ (0.10)	1.08–1.48
**Geographic area (East)**		
Central	0.82^*^ (0.08)	0.68–0.98
West	0.94 (0.10)	0.76–1.15
Educated	1.71^***^ (0.16)	1.42–2.05
Had Sufficient income	1.42^**^ (0.16)	1.14–1.76
Had chronic illness	1.16 (0.10)	0.97–1.38
Smoked	1.78^***^ (0.26)	1.33–2.38
Drank	1.81^***^ (0.27)	1.33–2.44
Took exercises	3.85^***^ (0.47)	3.04–4.88
Had medical insurance	1.10 (0.11)	0.90–1.33
Received adequate healthcare service	1.51^*^ (0.30)	0.97–1.38
R^2^	0.14	
Chi^2^	766.34^***^	

* *P < 0.05*,

** *P < 0.01*,

*** *P < 0.001*.

#### Robust test

First, we conducted the Durbin-Wu-Hausman test to verify that endogeneity existed between unmet needs and healthy aging levels (*p* < 0.01). Second, using the provincial old-age dependency ratio as the instrumental variable, we conducted both the 2SLS model and the CMP model to check the robustness of the regression analysis results. Findings were summarized in Table 5, showing that disabled older adults with unmet needs experienced the lower level of healthy aging than their counterparts with met needs (2SLS: *b* = −0.94, *SE* =0.35, *p* < 0.01; CMP: *b* = −1.19, *SE* =0.20, *p* < 0.001).

**Table 5:** Robust test of ordered logit regression analysis results (*N* = 2879).

	**2SLS model**		**CMP model**	
	b	SE	b	SE
Unmet needs	−0.94^**^	0.35	−1.19^***^	0.20
Control variables	Controlled			

** *P < 0.01*,

*** *P < 0.001*.

## Discussion

Existing research focused on the impact of unmet needs on the biomedical level of health. To our knowledge, this is the first study to measure healthy aging levels, and explore the relationship between unmet needs and different levels of healthy aging among Chinese older adults. Our results showed that most disabled older adults achieved the middle level of healthy aging while only < 10% achieved the high level of healthy aging, and that older adults with disabilities who experienced unmet needs for assistance had a lower probability of achieving the higher level of healthy aging than those with met needs.

Our study has implications for health research. Previous studies have established the relationship between unmet needs and a set of single health indicators which mainly represented the biomedical level of health, such as mortality, hospitalizations, rehospitalizations, and depression (11–17). Indeed, healthy aging comprises of physiological, psychological, and social domains. Therefore, more efforts are needed to explore the correlation between adequacy of LTC resources and overall health on a functional basis. This study is among the first to estimate the prevalence of Chinese disabled older adults experiencing different levels of healthy aging, which could set the basis for future studies to generate a harmonized index of healthy aging for international comparison. In addition, this is also the first study to understand the relationship between unmet needs for assistance and different levels of healthy aging. Since older adults with disabilities have complex health needs, a comprehensive measurement of healthy aging, rather than a single health outcome, could advance the knowledge on how the overall health would be affected by unmet needs, and it could set the basis for future studies to understand the relationship between unmet needs and the healthy aging index in aging populations.

This study highlights the need to take unmet needs for assistance as an indicator for assessing health needs and service quality. Although the Chinese LTC insurance pilot program has conducted a need assessment to identify older adults with severer disabilities as beneficiaries of publicly supported care, the disability criteria for benefit coverage typically overlooked the extent to which disability-related needs are met. Besides, evaluating the efficacy of LTC services is limited by the lack of appropriate outcome measures. Prior research indicated that more severe ADL limitations caused unmet ADL needs (19), which, in turn, might lead to a deterioration in healthy aging in this study, denoting the declining functioning of not only physical activities but also cognition and participation. And thus, a vicious spiral between unmet needs and declining functioning has been created, demonstrating the validity of unmet needs as an indicator for assessing health needs and service quality. Therefore, unmet needs could be a basis for risk assessment and a means for determining the efficacy of LTC interventions on maintaining health. If an older adult experiencing a met need changes to an unmet need, this might be a warning signal that the older adult is at a higher risk of losses in health. In contrast, if the changes occur in the opposite way, he or she is more likely to have ameliorated losses and achieve healthy aging.

This research increases the awareness of gerontological practitioners to the LTC services and supports for disabled older adults in China, which is shown to enhance their healthy aging in a developing country with the largest older adult population globally. Medical diagnosis and treatment, which are of high cost and require professionalism, have been the main means for maintaining health based on the traditional biomedical model. Compared to the single diagnosis and treatment that simply reacts to specific disease individually, LTC services can manage the complex needs of older age in an integrated way. Moreover, considering that the trajectory of functional decline is irreversible, LTC services will be more effective in not just delaying the process of decline, but ensuring disabled older adults have more opportunities for participation. And thus, the provision of sufficient and high-quality LTC services and supports for disabled older adults seems to be an effective intervention for improving healthy aging.

We note several limitations of this study. First, due to data restriction, this study was unable to examine the association between different types of unmet needs for assistance and healthy aging. Prior research identified differences between unmet needs for assistance with ADLs and IADLs (19), and also distinguished subjective unmet needs from objective unmet needs (55). Future studies could further explore different types of unmet needs for assistance and their influences on healthy aging if data become available. Second, our study is among the first to identify the latent class of older adults experiencing different levels of healthy aging by using three observed indicators, including ADLs, cognition, and participation. Future empirical studies are needed to add the social adaption and the age-friendly environments, another two fundamental indicators of healthy aging, to generate the latent classes of healthy aging. Lastly, our analysis included only community-dwelling older adults. Nursing home residents are, therefore, not represented in this analysis. Given the higher prevalence of functional impairment and other health problems in nursing home residents, our estimates of the percentage of older adults experiencing the low level of healthy aging are undoubtedly higher than their counterparts in nursing homes.

## Data availability statement

Publicly available datasets were analyzed in this study. This data can be found here: https://opendata.pku.edu.cn/dataset.xhtml?persistentId=10.18170/DVN/WBO7LK.

## Ethics statement

The studies involving human participants were reviewed and approved by the Ethics Review Committee of Duke University and Peking University (IRB00001052e13074). The patients/participants provided their written informed consent to participate in this study.

## Author contributions

YC and YL designed the study, edited the manuscript and supervised the data analysis. YC and YF drafted the first version of the article. YF performed all statistical analyses. All authors have read and approved the manuscript.

## Funding

This work was supported by the National Social Science Fund of China [Grant number 21CRK003].

## Conflict of interest

The authors declare that the research was conducted in the absence of any commercial or financial relationships that could be construed as a potential conflict of interest.

## Publisher's note

All claims expressed in this article are solely those of the authors and do not necessarily represent those of their affiliated organizations, or those of the publisher, the editors and the reviewers. Any product that may be evaluated in this article, or claim that may be made by its manufacturer, is not guaranteed or endorsed by the publisher.

## Abbreviations

ADLs: Activities of Daily Living
AIC: Akaike's Information Criterion
aBIC: adjusted Bayesian Informal Criterion
BIC: Bayesian Informal Criterion
CLHLS: the Chinese Longitudinal Healthy Longevity Survey
CMP: Conditional Mixed Process Model
IADLs: Instrumental Activities of Daily Living
LMR-LRT: Lo-Mendell–Rubin Likelihood Ratio Test
LPA: Latent Profile Analysis
LTC: Long Term Care
MMSE: Mini Mental State Examination scale
SE: Standard Error
WHO: World Health Organization
2SLS: Two Stage Least Squares.
